# The protein composition of exosomes released by prostate cancer cells is distinctly regulated by androgen receptor-antagonists and -agonist to stimulate growth of target cells

**DOI:** 10.1186/s12964-024-01584-z

**Published:** 2024-04-08

**Authors:** Golnaz Atri Roozbahani, Miriam Kokal-Ribaudo, Mehdi Heidari Horestani, Thanakorn Pungsrinont, Aria Baniahmad

**Affiliations:** Institute of Human Genetics, Jena University Hospital, Am Klinikum 1, 07740 Jena, Germany

**Keywords:** Prostate cancer, Androgen receptor agonist, Antagonist, Cellular senescence, Supraphysiological androgen levels, Extracellular vesicles, Exosomes

## Abstract

**Background:**

Prostate cancer (PCa) is a prevalent malignancy in men worldwide, ranking as the second leading cause of cancer-related death in Western countries. Various PCa hormone therapies, such as androgen receptor (AR)-antagonists or supraphysiological androgen level (SAL) reduce cancer cell proliferation. However, treated cells may influence the growth of neighboring cells through secreted exosomes in the tumor microenvironment (TME). Here, the change of protein content of exosomes secreted from PCa cells through treatment with different AR-antagonists or SAL has been analyzed.

**Methods:**

Isolation of exosomes via ultracentrifugation of treated human PCa LNCaP cells with AR-agonist and various AR-antagonists; analysis of cellular senescence by detection of senescence associated beta galactosidase activity (SA β-Gal); Western blotting and immunofluorescence staining; Mass spectrometry (MS-spec) of exosomes and bioinformatic analyses to identify ligand-specific exosomal proteins. Growth assays to analyze influence of exosomes on non-treated cells.

**Results:**

MS-spec analysis identified ligand-specific proteins in exosomes. One thousand seventy proteins were up- and 52 proteins downregulated by SAL whereas enzalutamide upregulated 151 proteins and downregulated 42 exosomal proteins. The bioinformatic prediction indicates an up-regulation of pro-proliferative pathways. AR ligands augment hub factors in exosomes that include AKT1, CALM1, PAK2 and CTNND1. Accordingly, functional assays confirmed that the isolated exosomes from AR-ligand treated cells promote growth of untreated PCa cells.

**Conclusion:**

The data suggest that the cargo of exosomes is controlled by AR-agonist and -antagonists and distinct among the AR-antagonists. Further, exosomes promote growth that might influence the TME. This finding sheds light into the complex interplay between AR signaling and exosome-mediated communication between PCa cells.

**Supplementary Information:**

The online version contains supplementary material available at 10.1186/s12964-024-01584-z.

## Background

Prostate cancer (PCa) is the most prevalent form of solid cancer among men. In spite of the impressive endeavors in screening and diagnostic protocols, PCa remains the second leading cause of cancer-related death in male in Western countries [1]. PCa is under regulation of androgen through the androgen receptor (AR) signaling [2]. Consequently, the primary objective of initial therapeutic hormonal interventions is to impede AR signaling via androgen deprivation therapy (ADT) either alone or in combination with AR-antagonists, leading to an initial regression of the cancer. Eventually the cancer becomes resistant due to adaptive responses of AR-signaling and activation of other signaling mechanisms [3, 4]. Bicalutamide (Bic), as the first-generation of AR-antagonists, and the subsequent second-generation of AR-antagonists, Enzalutamide (Enz) and Darolutamide (Dar), are established clinical interventions employed to block AR activity in PCa [5]. Besides these AR-antagonists, Atraric acid (AA) is the first natural compound identified and characterized as an AR-antagonist, suppressing PCa cell growth in cell culture, 3D spheroids and in xenograft mice [6, 7].

In addition to AR-antagonists, androgen levels exert a significant influence on the growth of PCa [8]. Treatment of PCa cells or patients with testosterone concentrations equivalent to adult male physiological levels, promote cell proliferation and tumor progression. However, exposure to supraphysiological androgen levels (SAL) leads paradoxically also to growth suppression of PCa [9, 10]. This intriguing approach, known as bipolar androgen therapy (BAT), using ADT conditions with cycling administration of testosterone, leading to altering periods of very low androgen levels and SAL [10–12]. BAT has been clinically investigated and shows promising results in clinical phase II trial [13].

Besides AR-antagonists, SAL induces cellular senescence in PCa cells as well as PCa samples derived from patients [14–16]. Cellular senescence represents a durable cessation cell cycle arrest [17]. In general, senescent cells are metabolically active and can influence a tumor microenvironment (TME) by releasing factors that can either promote or suppress tumor development [18]. Within the TME, tumor cells engage in local and long-range signaling with various neighboring cells [19]. Senescent cells secreted factors known as senescence-associated secretory phenotype (SASP) including inflammatory cytokines, chemokines and growth factors as well as exosomes [16, 20, 21].

Exosomes are small extracellular vesicles surrounded by a lipid bilayer membrane and released by the majority of eukaryotic cells with exosome marker such as CD9 [22]. Exosomes facilitate intercellular communication and modulation of biological processes of target cells [22]. The specific biological function of exosomes depend on their cargo [23]. Tumor cells from different origins have been found to produce and release exosomes to promote tumor growth, making them an important issue for tumorigenesis, proliferation, survival, migration and drug resistance [22]. Emerging research highlights the significance of intercellular communication facilitated by exosomes in the progression and metastasis of PCa [24, 25]. Therefore, exosomes have the potential to serve as diagnostic and prognostic markers [22].

The hypothesis is that AR targeted therapies of PCa may influence exosome secretion and the composition of exosome cargo. Here, we addressed whether supraphysiological androgens or various AR-antagonists influence the protein content of exosomes. To the best of our understanding, no prior research analyzed the differentially presence of factors of PCa exosomes dependent on AR-ligands treatment. Here we isolated exosomes from LNCaP cells treated with AR-antagonists, including Enz, AA and Dar, as well as SAL. Subsequently, we employed MS-spec and bioinformatics predictions. The data suggest an enrichment of growth promotion, membrane activity and neural pathways. Functionally, we confirmed that exosomes lead to enhanced growth of naïve LNCaP cells.

## Materials and methods

### Cell culture and treatments

LNCaP cells [26], were cultured in RPMI-1640 medium (Gibco Life Technologies) supplemented with 5% fetal bovine serum (FBS), 25 mM HEPES pH 7.5, 100 U/ml penicillin, 100 μg/mL streptomycin, and 1% sodium pyruvate. LNCaP were seeded in cell culture plates and in a 5% CO_2_, humidified atmosphere at 37 °C. Following a 48 h incubation period, the cells were treated for 72 h with 1 nM R1881 (SAL), 1 μM Enzalutamide (Enz), 10 μM Darolutamide (Dar), 1 μM Bicalutamide (Bic), 100 μM Atraric Acid (AA) or 0.1% DMSO as a solvent control (C).

### Senescence-associated β-galactosidase (SA β-gal) staining

For SA β-gal staining assay 35,000 LNCaP cells per well in 6-well plates were seeded. SA β-gal staining and detection were performed as described previously [15, 27].

### Methanol precipitation of secreted proteins

500,000 LNCaP cells per 10 cm dish were seeded and treated for 72 h with DMSO, SAL, Enz, AA, Bic, or Dar. The conditioned medium was collected after 48 h of incubation with 0% FBS medium. To the collected conditioned medium, a nine-fold medium volume of cold 100% methanol was added, thoroughly mixed and incubated on ice and centrifuged at 3700 g for 20 min at 4 °C. The pellets were dissolved in cold 90% methanol and centrifuged at 15,000 g for 15 min at 4 °C. The pellet was dissolved in 100 μl Milli-Q H_2_O water.

### Western blotting and antibodies

Protein samples were loaded and separated by SDS polyacrylamide gel. The membrane was incubated with specific primary antibodies against PSA (Cell Signaling, 24,755) and ANG (Boster, A00146). Horseradish peroxidase-conjugated anti-mouse IgG (Cell Signaling, 7076S) or anti-rabbit IgG (Cell Signaling, 7074S) were used as secondary antibodies. Finally, signals were detected by ImageQuant™ LAS 4000 (GE Healthcare Bio-Sciences AB) using ECL reagents (GE Healthcare). Quantification of bands were performed via the LabImage D1 program.

### Immunofluorescence staining

15,000 LNCaP cells were seeded on coverslips in 24-well plates. After 48 h, cells were treated with DMSO, SAL or Enz. Following a 72 h of incubation, the cells were washed three times with 1x PBS and then fixed with 4% paraformaldehyde for 15 min at room temperature. Following a wash with 1x PBS, the cells were permeabilized using 0.25% Triton X. After two additional washes, the cells were blocked with 5% normal goat serum for 1 h at room temperature. Subsequently, the cells were incubated overnight at 4 °C with primary antibodies against CD9 (Invitrogen, 10626D) and TIMP2 (ABclonal, A1558). The next day, cells were washed and incubated with the secondary antibodies for 1 h at room temperature in a dark room. To visualize the nuclei, the cells were incubated with Hoechst (1:10,000, Invitrogen, H3569) for 5 min, followed by another wash with 1x PBS. The coverslips were transferred to glass slides using Fluoromount G (BIOZOL, SBA-0100-01). The slides were then dried, and pictures were captured with a Zeiss LSM 880 with Airyscan scanning fluorescence microscope equipped with a Plan-Apochromat 63x/1.4 oil DIC M27 objective at super resolution. Fiji software [28] was used for the quantification. The intracellular protein level was defined by the normalized total cell fluorescence (NTCF) set as relative to DMSO (Eq. 1). The secretion of exosomes was defined by Eq. 2 set as relative to DMSO.

1 $$NTCF= IntDen-\left( Area\ of\ selected\ cell\ast Mean\ fluorescence\ of\ BR/n\right)$$

NTCF: normalized total cell fluorescence, IntDen: Integrated Density, BR: background readings

2 $$m ean\ secreted\ intensity/\mu m2= RInt\ total- RInt\ cells/ Area\ total- Area\ cells$$

RInt: Raw Intensity.

### Exosome isolation from conditioned medium

500,000 LNCaP cells per 10 cm dish were seeded and subjected to treatment with DMSO, SAL, Enz, AA, or Dar for 72 h. Each treatment was performed in four dishes. The conditioned medium was collected after 48 h of incubation with 0% FBS medium. Exosomes were isolated from LNCaP conditioned medium using the differential centrifugation protocol. Briefly, conditioned medium was centrifuged at 380 g for 10 min at 4 °C to sediment cells and at 10,000 g for 10 min to eliminate cell debris. Consequently, the supernatant was centrifuged at 18,900 g for 30 min at 4 °C to remove microparticles and contaminating proteins. Exosomes were sedimented by two times ultracentrifugation at 100,000 g for 75 min at 4 °C and pellets were resuspended either in serum-free medium for growth assay or PBS for MS-spec.

### Proteomics analysis by MS-spec

Exosomes were collected, proteins reduced, alkylated and precipitated with 8x volumes of cold acetone (Biosolve, #010306), as described elsewhere [29]. Precipitated proteins were resuspended in digestion buffer containing 1 M Guanidine HCl (Roth, 0035.1) in 100 mM HEPES (Sigma, H3375-100G) pH 8.0 and digested for 4 h at 37 °C using 1:100 (w/w) LysC (Wako Chemicals GmbH, #125–05061). Then, samples were diluted to 0.5 M Guanidine HCl with MilliQ water and digested with 1:100 (w/w) trypsin (Promega, #V5111) for 16 h at 37 °C. Digested peptide solutions were then acidified with 10% (v/v) trifluoroacetic acid and then desalted with Waters Oasis® HLB μElution Plate 30 μm (Waters, 186001828BA) in the presence of a slow vacuum, following manufacturer instructions. Eluates were dried with a speed vacuum centrifuge and dissolved in 5% (v/v) acetonitrile, 0.1% (v/v) formic acid to a peptide concentration of ~ 1 μg/μl, transferred to a MS vial and spiked with iRT peptides (Biognosys AG, Ki-3002) prior to analysis by LC-MS.

Approx. 1 μg of digested peptides were analyzed by Data Independent Acquisition (DIA) using the M class UPLC system (Waters) with a trapping (nanoAcquity Symmetry C18, 5 μm, 180 μm × 20 mm) and an analytical column (nanoAcquity BEH C18, 1.7 μm, 75 μm × 250 mm). Coupled to a Q exactive HF-X (Thermo Fisher Scientific) using the Proxeon nanospray source, as described elsewhere [30]. The raw files were processed by directDIA analysis using Spectronaut Professional+ v13.10 (Biognosys AG). Raw files were searched by directDIA search with Pulsar (Biognosys AG) against the human UniProt database (*Homo sapiens*, reviewed entries only, release 2016_01) with a list of common contaminants appended, using default settings. For quantification, default BGS factory settings were used, except: Proteotypicity Filter = Only Protein Group Specific; Major Group Quantity = Median peptide quantity; Major Group Top N = OFF; Minor Group Quantity = Median precursor quantity; Minor Group Top N = OFF; Data Filtering = Qvalue; Normalisation Strategy = Local normalisation; Row Selection = Automatic. The candidates and report tables were exported from Spectronaut and used for further analysis. Protein groups were considered as significantly affected if they displayed a Q value < 0.05.

### Growth assays

A total of 13,000 LNCaP cells were seeded in each well of 6-well plates. After 48 h, the absorbance of crystal violet staining was measured on two wells from each treatment, representing day 0. The remaining wells were treated with isolated exosomes derived from 2,000,000 cells. Isolated exosomes were resuspended in serum-free medium and diluted 1:1 with fresh medium containing 10% FBS. Over the course of six days, the absorbance of crystal violet was measured every two days on two wells from each treatment, while the medium containing isolated exosomes was refreshed in the remaining wells. Subsequently, the actual absorbance was calculated relative to the absorbance of DMSO on day 0.

### Bioinformatics analysis

Pathway analysis was conducted using pathfindR package [31, 32] with a significant threshold set at *p* < 0.05 to filter significant proteins. Protein sets were defined according to Reactome database and protein-protein interaction network was defined according to Biogird. Additionally, for certain aspects of pathway analysis, the Enrichr webtool was employed [33–35].

### Statistical analysis

Graph Pad Prism 8.0 software was utilized for statistical analysis. The data were expressed as the mean ± SD and were derived from a minimum of three independent experiments. Statistical significance for each experiment was determined using the appropriate method, either a two-tailed unpaired t-test or two-way analysis of variance (ANOVA).

## Results

### The level of secreted factors is dependent on AR ligands

Prostate specific antigen (PSA) is well known as a diagnostic marker for PCa, which is upregulated by androgens and secreted by LNCaP cells. To analyze the activity of AR-ligands, including the first-generation antagonist Bic, the second-generation AR-antagonists Enz and Dar, and the natural AR-antagonist AA, we analyzed the secretion of PSA in the supernatant cell culture medium by Western blotting (Supplementary Fig. S1). In contrast to dihydrotestosterone, which is rapidly metabolized and its metabolites may act as estrogen receptor beta agonists [36], the much less metabolizable synthetic androgen methyltrienolone (R1881) and thus more AR-specific androgen was used at 1 nM, defined previously as SAL [14, 15]. DMSO was used as solvent control. As expected, the data suggest upregulation of PSA secretion by SAL (Supplementary Fig. S1).

**Fig. 1:**
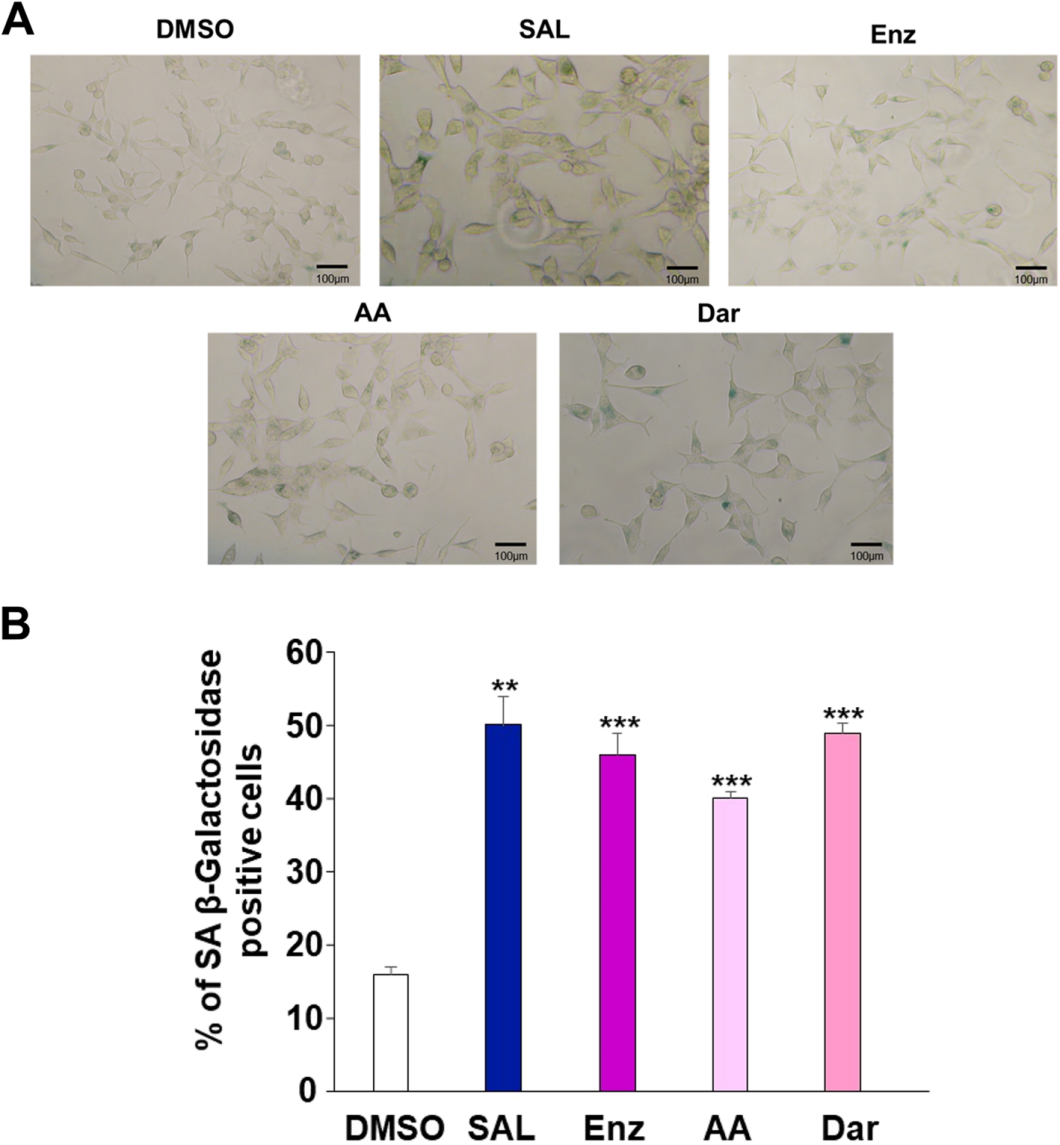
AR-antagonists induce cellular senescence in LNCaP cells. **A** SA β-Gal activity staining of LNCaP cells were visualized under a light microscope (magnification 100 x). **B** Bar chart indicates the percentage of SA β-Gal positive LNCaP cells. The mean ± SEM values were calculated from the three independent experiments (*n* = 3). A two-tailed unpaired Student’s t-test was performed for statistical analysis (stars indicate a statistical significance with *p < 0.05, **p < 0.01, ***p < 0.001)

Previously, it was shown that treatment with AR-antagonists induces cellular senescence [20, 37, 38]. Similarly, using AR-agonist at SAL cellular senescence is induced in PCa cell lines and in patient samples treated ex vivo [14, 15, 39]. Here, we confirmed the induction of cellular senescence in LNCaP cell line, using SAL, Enz, or AA, and show also induction of cellular senescence by Dar treatment observed after 72 hours of AR-ligand treatment (Fig. 1 A). The percentage of SA β-Gal positive stained cells as a marker of cellular senescence was quantified (Fig. 1 B). The data suggest that the used AR-antagonists as well as SAL treatment significantly induce cellular senescence in LNCaP cells.

Angiogenin, ANG, is known to be secreted by human PCa and contributes to cancer progression through mediating tumor angiogenesis, cancer cell survival, and proliferation [40]. To confirm that AR-ligand induced senescent cells secrete ANG, the conditioned medium of treated cells was collected and ANG was detected by Western blotting (Supplementary Fig. S1). It is hypothesized here that the secretion of ANG is distinctly regulated dependent on the treating of a specific AR-ligands [41]. The secretion levels of ANG were upregulated upon SAL treatment, while AR-antagonists reduced ANG levels in the medium but in a distinct manner, with AA reducing only weakly, whereas Bic, Enz, and Dar repress the secreted ANG-level more potently. These findings suggest that AR-ligands influence the secretion of factors.

### CD9 levels are regulated by SAL

**Fig. 2:**
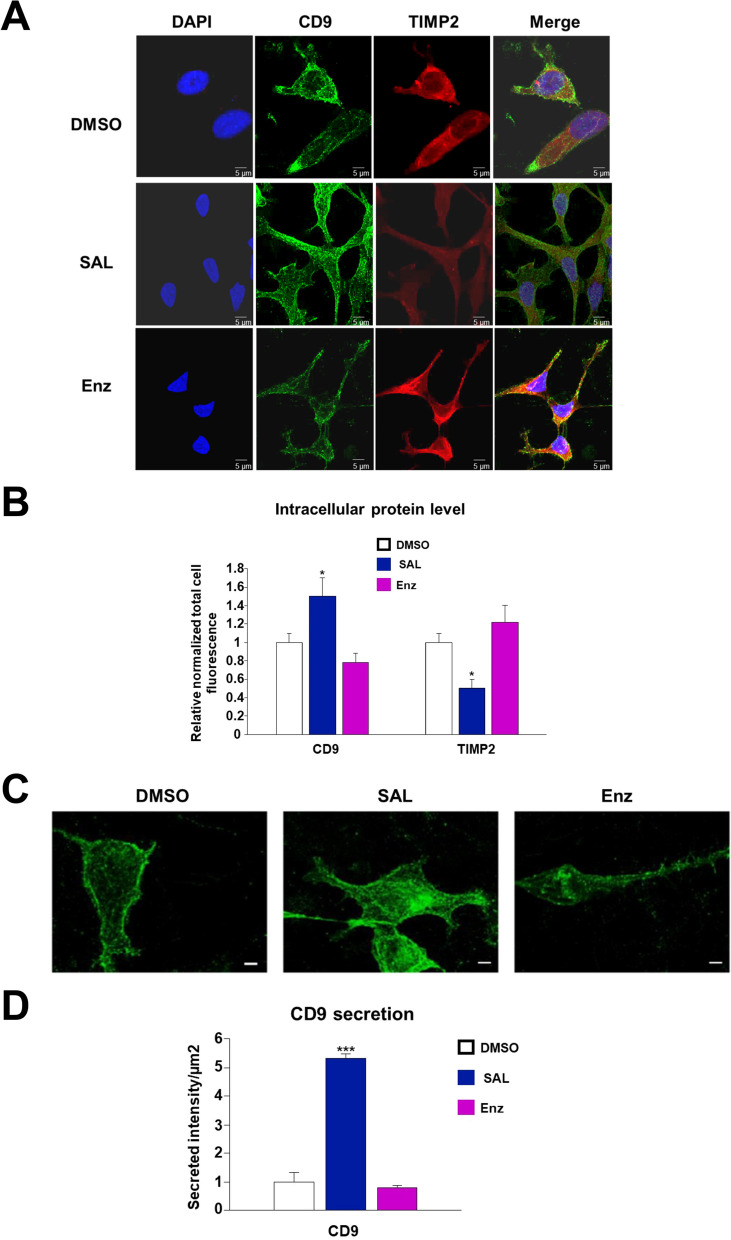
Androgen treatment increases CD9 protein level and induces CD9 secretion. **A** Fluorescent intensity measurement with the LSM. Nucleus (blue), CD9 (green), TIMP2 (red). Scale bars indicate 5 μm. **B** The intracellular protein level was defined as the normalized total cell fluorescence set as relative to DMSO. The mean ± SEM values were calculated from the technical replicates (*n* = 15). A two-tailed unpaired Student’s t-test was performed for statistical analysis (*p < 0.05, **p < 0.01, ***p < 0.001). **C** The secretion of CD9 from the same cells using signals in (A) detected outside of cells was measured using the LSM. Scale bars indicate 5 μm. **D** The secreted intensity of CD9 was determined. The mean ± SEM values were calculated from the technical replicates (n = 15). A two-tailed unpaired Student’s t-test was performed for statistical analysis (*p < 0.05, **p < 0.01, ***p < 0.001). CD9 (cluster of differentiation 9), LSM (laser scanning microscope)

Since ANG is known to be a key factor in exosomes [42, 43], we focused on the AR-ligand control secretion of exosomes. Also, CD9 is a key factor of exosomes located on their membrane and serves as an exosome marker, associated with cancer migration and invasion [44–46]. To analyze the AR-agonist and -antagonist regulation of intracellular levels of CD9, immunofluorescence staining were performed and images acquired with a laser scanning microscope (LSM). LNCaP cells were treated with SAL, Enz or DMSO as solvent control (Fig. 2 A). In addition to CD9 immunostaining, TIMP2 immunostaining was included. TIMP2 is known to be a SASP factor [47] and shown to function in tumor suppression [48] and has not yet been identified in exosomes. TIMP2 exhibits rather a non-punctuated distribution in the cytosol. Following SAL treatment, TIMP2 levels decreased (Fig. 2 A, B). In contrast, CD9 was detected in a punctuated-speckled distribution predominantly localized in the cytosol and at the plasma membrane. Interestingly, intracellular CD9 levels were increased by SAL and decreased by Enz treatment (Fig. 2 A, B). This suggests that AR-ligands may not regulate the secretion of CD9 exosomes rather the intracellular protein levels of CD9. The data also indicate that TIMP2 may not co-localize with exosomes.

Further, we analyzed CD9 signals secreted from LNCaP cells. The secretion was analyzed upon AR-ligand treatments (Fig. 2 C and D). The data suggest that CD9 secretion significantly increased after SAL treatment, while no significant changes were observed following Enz treatment. These results indicate that androgen increased intracellular CD9 protein levels and enhanced the CD9 secretion, implying that exosome levels are controlled by AR-ligands in LNCaP cells.

Our findings suggest that SAL-activated AR enhances CD9 levels and indicate enhanced secretion of exosomes. Of note, the AR-antagonist Enz also induces cellular senescence (Fig. 1 A and B) but seem not to enhance CD9 levels.

### SAL and Enz change the protein content of exosomes

**Fig. 3:**
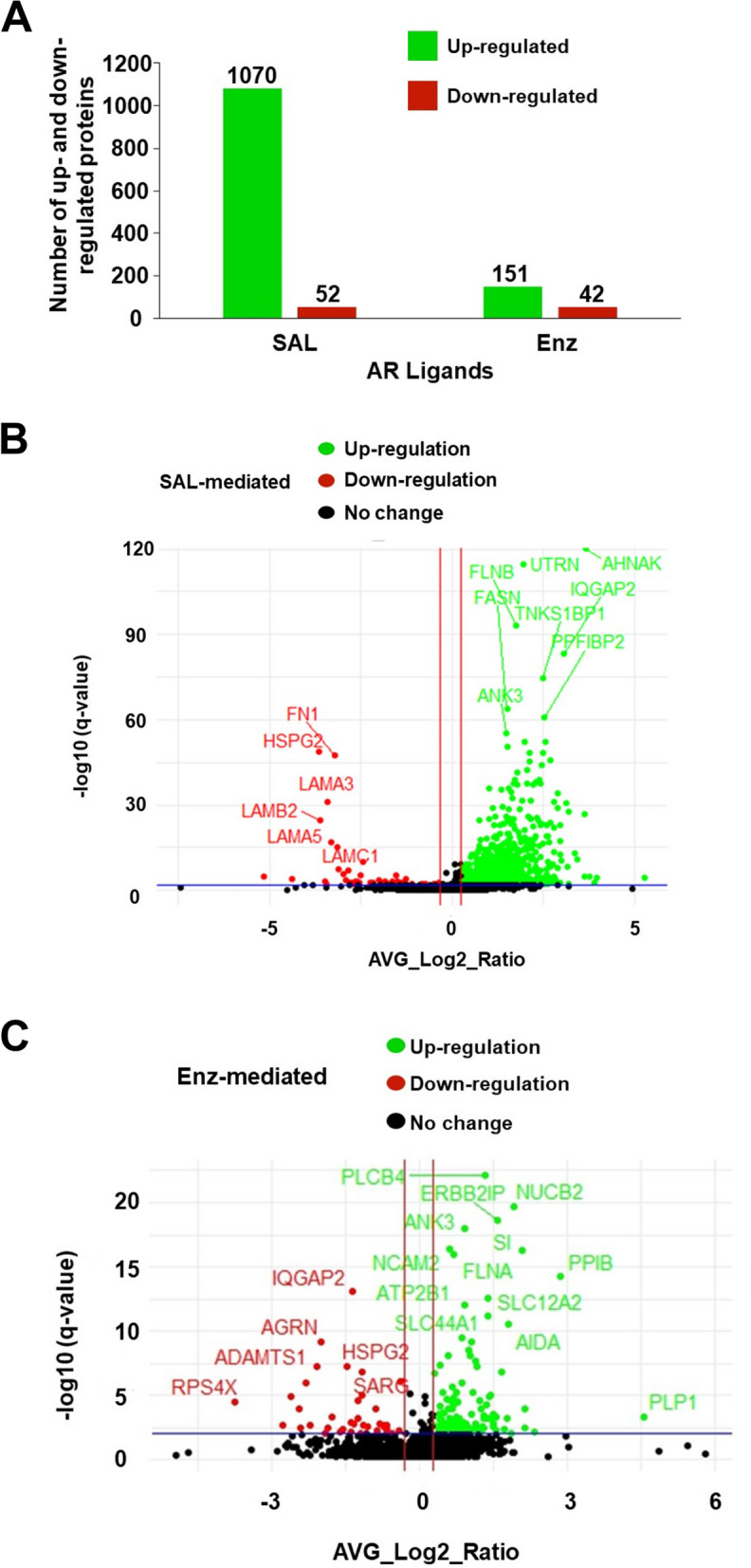
Significant up- or downregulated exosomal proteins by SAL and Enz. **A** Bar chart shows up and downregulated proteins (*n* = 4). **B-C** Volcano plots show the differentially expressed exosomal proteins. Proteins that were not classified as up- or downregulated are represented in black color (B: SAL vs. DMSO; C: Enz vs. DMSO; *p* < 0.05; *n* = 4). Red vertical lines define fold change ≥0.2

To identify factors within the exosomes and analyze whether AR-agonist and AR-antagonists affect the exosome protein content, MS-spec was employed. Cells were treated with the AR-antagonist Enz and androgen at SAL for 72 h. The culture medium was replaced with fresh serum-free medium for an additional 48 h. The supernatant was collected, removed from cell debris and exosomes were then isolated from this conditioned medium by ultracentrifugation. Following MS-spec analysis, a total of 2267 proteins were identified. The MS-spec data revealed changes in the exosomal protein content in response to SAL treatment. 1070 significantly upregulated and 52 significantly downregulated proteins were identified by SAL treatment compared to the solvent control DMSO (Fig. 3 A; Supplementary Table S1). Furthermore, a significant alteration in the exosomal protein content in response to Enz treatment compared to DMSO was detected, with 151 proteins being upregulated and 42 proteins downregulated (Fig. 3 A; Supplementary Table S2). The different expression of exosomal proteins following treatment with SAL and Enz is visually represented in the volcano plots (Fig. 3 B and C). The criteria for protein selection in these plots were of fold change ≥1.2 or fold change ≤0.8 and *p* < 0.05. Highlighted within these plots are proteins with key roles in important cellular processes and signaling pathways. For instance, proteins upregulated by SAL (Fig. 3 B) are associated with promoting cell growth, intracellular signaling, or cellular communication within the tumor microenvironment. E.g. AHNAK is known to play a role in cell adhesion and migration [49]. Conversely, the SAL-mediated downregulated proteins (Fig. 3 B) may be involved in suppressing certain pathways or function in exosomes. These proteins are associated with the regulation of ECM remodeling. Regarding Enz treatment, the upregulated proteins (Fig. 3C) play roles in enhancing cellular process potentially associated with calcium signaling pathway. On the other hand, the downregulated proteins (Fig. 3 C) may participate in pathways related to cell adhesion. These shift in protein composition can potentially impact cellular process such as tumor growth, angiogenesis, and metastasis.

This data suggests that SAL and Enz treatment specifically change the protein content of exosomes and provide an insight into AR regulation of exosomal proteins.

### Bioinformatic pathway analysis of exosomal proteins derived from SAL and Enz treatments predicts tumor promoting activity

**Fig. 4:**
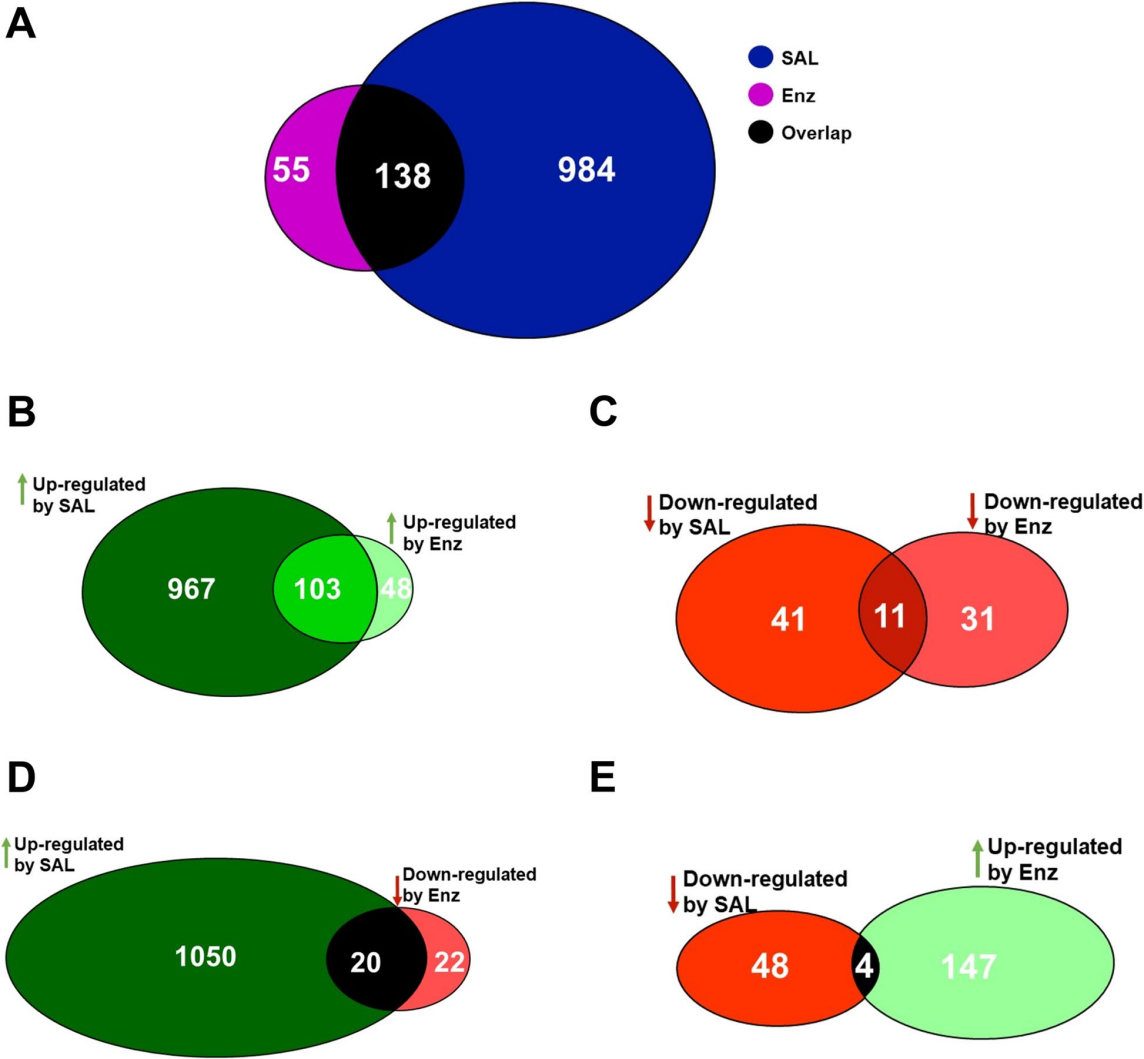
138 common proteins were detected between SAL and Enz treated LNCaP cells in exosomes. **A** Venn diagram shows overlapped proteins between SAL and Enz (*n* = 4). **B** 103 common upregulated proteins between SAL and Enz. **C** 11 common downregulated proteins between SAL and Enz. **D **20 proteins upregulated by SAL and downregulated by Enz. **E** 4 proteins downregulated by SAL and upregulated by Enz. (*n* = 4)

The level of proteins changed by AR-ligand treatment were further analyzed bioinformatically. Overlaps of up- and downregulated proteins with similar or opposite regulation by SAL and Enz were further characterized (Fig. 4). Among the differentially present proteins, 984 factors are specifically regulated by SAL, and 55 factors are regulated by Enz. Interestingly, although based on different factors some similar signaling pathways were identified in the bioinformatic analysis by both treatments including RHO GTPase cycle, RHOU GTPase cycle, cellular response to starvation, signaling by EGFR, signaling by MET, and apoptotic cleavage of cell adhesion proteins. This suggest that differentially secreted proteins have overlapping pathways, prompting further investigation into common factors in the microenvironment (Supplementary Table S3 and Table S4). 138 commonly regulated exosomal proteins between SAL and Enz treatment were identified (Fig. 4 A). Among these, 103 were upregulated (Fig. 4 B; Supplementary Table S5) and 11 were downregulated (Fig. 4 C; Supplementary Table S6) by both treatments. Furthermore, 20 of the 138 proteins exhibited an upregulation by SAL treatment but downregulation after Enz treatment (Fig. 4 D; Supplementary Table S7), while 4 of the 138 proteins displayed a downregulation in response to SAL but an upregulation by Enz treatment (Fig.4 E; Supplementary Table S8).

**Fig. 5:**
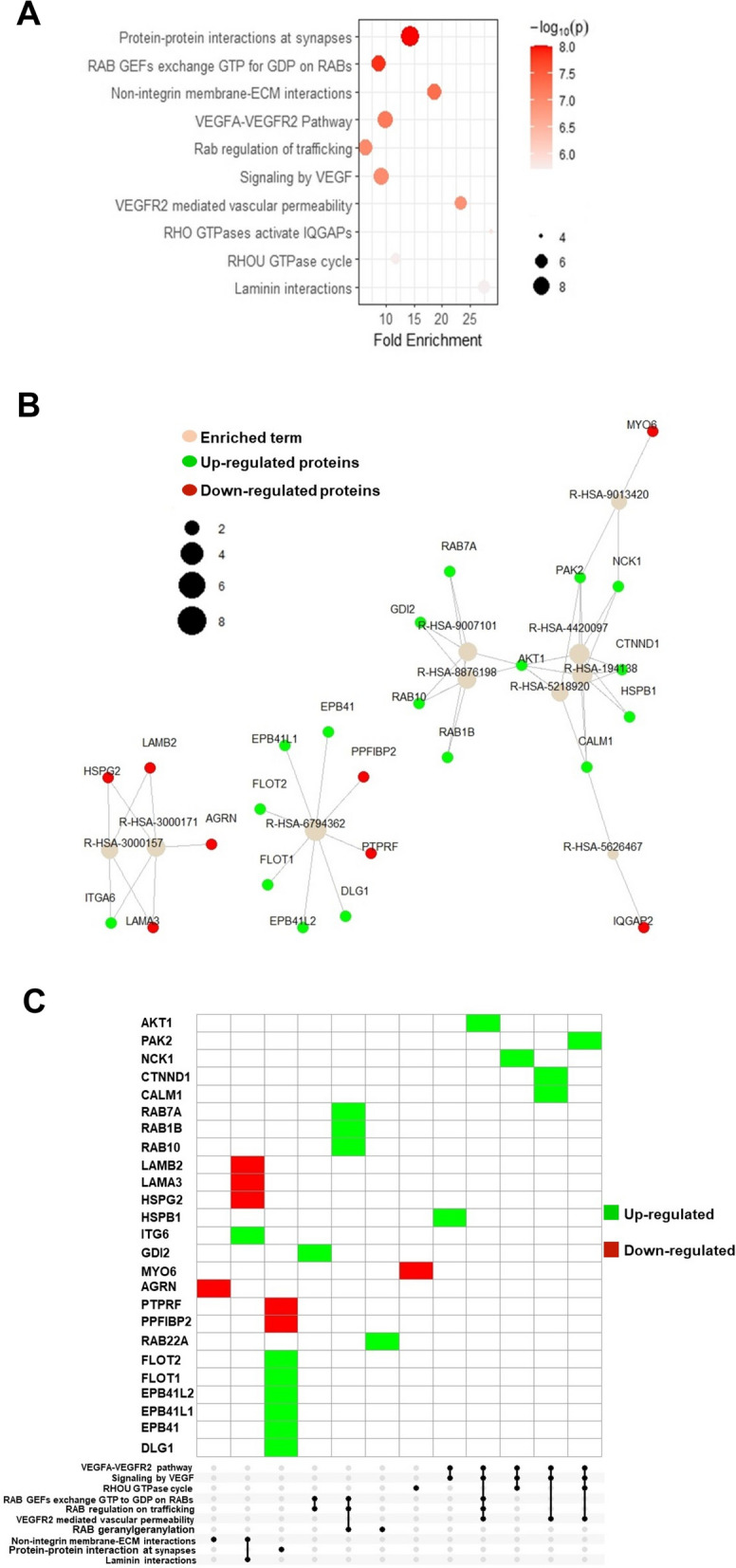
Pathway analysis of 138 common exosomal proteins suggest activation of pro-proliferative pathways. **A** Highest enriched pathways according to the detected 138 common exosomal proteins between SAL and Enz. X-axis represents the fold enrichment of the expressed proteins enriched in the indicated pathway. Size of the dots indicates the number of significant proteins in the given enriched pathway. Color indicates -log10 (lowest *p*-value). **B** Network visualizes which proteins are involved in the enriched pathway and how the proteins are connected in different pathways. Pathways in the network are shown according to the reactome ID number. **C** UpSet Plot shows a matrix of enriched pathways and the number of proteins at the corresponding intersections of enriched pathways. (*n* = 4)

Pathway analysis using PathfindR [31] was performed for these 138 common proteins to predict features of these exosomal factors in response to AR-ligands. Top 10 predicted significantly enriched signaling pathways indicate an upregulation of pro-cancerogenic pathways (Fig. 5 A; Supplementary Fig. S2A, S3A, S4A). Additionally, network interactions within these pathway-involved proteins were examined for each pathway. These illustrate the involvement of individual proteins in various pro-cancerogenic signaling pathways. For instance, CALM1 activates four pathways, VGFA-VEGFR2 pathways (R-HSA-4420097), signaling of VEGF (R-HSA-194138), VEGFR2-mediated vascular permeability (R-HSA-5218920), and RHO GTPases activate IQGAPs (R-HSA-5626467) (Fig. 5 B; Supplementary Fig. S2B). Network interactions analysis was performed for the common proteins that were either upregulated or downregulated by SAL and Enz (Supplementary Fig. S2B, S3B, S4B). UpSet plot showed the five major pathways, including VEGFA-VEGFR2 pathway, signaling by VEGF, RAB GEFs exchange GTP to GDP on RABs, RAB regulation on trafficking and VEGFR2 mediated vascular permeability, are encoded by upregulated proteins, such as AKT1, CTNND1, HSPB1 (Fig. 5 C; Supplementary Fig. S2C). This suggests an enrichment of proteins activate tumor growth by both treatments. The 11 downregulated proteins in both treatments are involved in membrane activity pathways (Supplementary Fig. S3C). Additionally, two separate UpSet plots were depicted for the 20 upregulated exosomal proteins following SAL treatment but were downregulated after Enz treatment (Supplementary Fig. S4C).

**Fig. 6:**
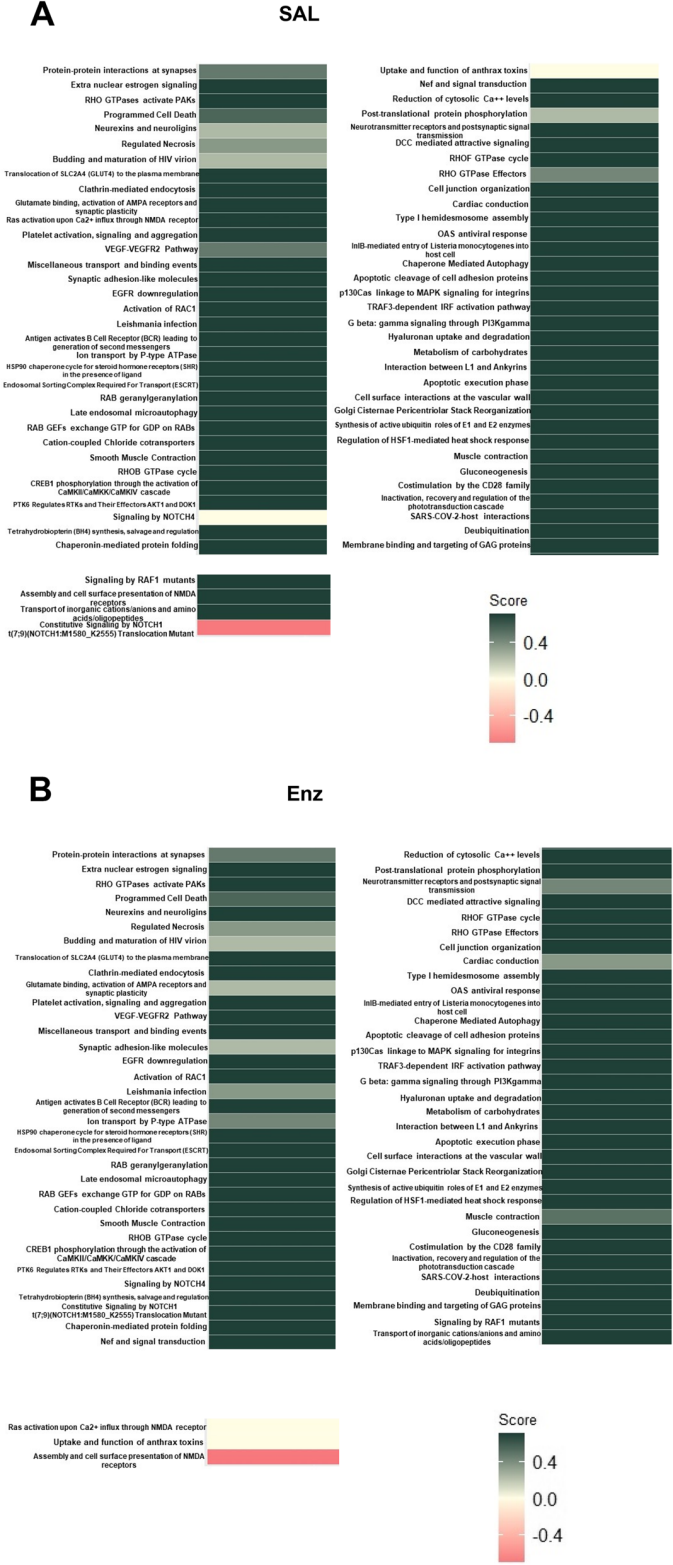
The content of exosomes from LNCaP cells treated with SAL and Enz, are mainly involved in growth promoting, membrane activity and neural pathways. **A-B** Overall alteration (activated or repressed) pathways based on 103 common upregulated proteins between SAL (**A**) and Enz (**B**) (each *n* = 4)

To assess the overall alteration of common pathways between SAL and Enz, we performed “aggregated term score per sample” analysis and visualized the results in a heatmap plot (Fig. 6). This analysis indicates the involvement of approximately 19 pathways for growth promotion, 14 signaling pathways are related to enhanced membrane activity, and 6 pathways are associated with neural pathways (Fig. 6 A and B).

Collectively, these findings provide insights into the regulation of AR-ligand controlled exosome cargos derived from treated LNCaP PCa cells and predict tumor promoting activity.

### Identified hub-proteins and pathway analysis of exosomal proteins common among AR-ligands indicate activation of pro-proliferation pathways

**Fig. 7:**
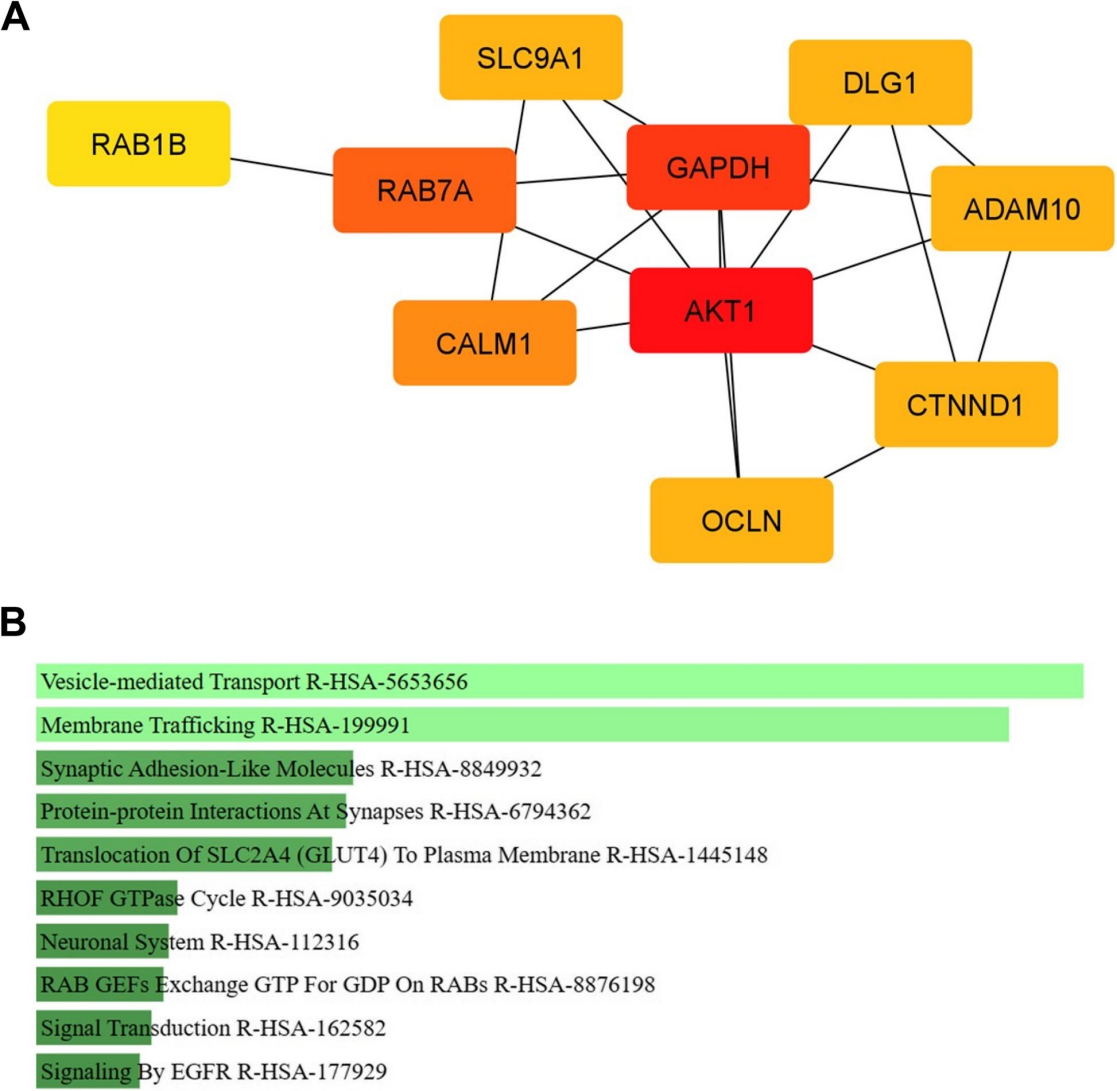
Enriched pathways of 83 common exosomal proteins among SAL, Enz, AA or Dar treatments. **A** Hub-proteins network of top 10 common exosomal proteins among SAL, Enz, AA or Dar treatments. Network was drawn by cytoscape [50]. The colors represent the rank of proteins, with red indicating a higher rank and yellow indicating a lower rank. The network was calculated based on the Maximal Clique Centrality (MCC) score. **B** The 10 most significantly enriched pathways according to Enrichr web tool (*n* = 4). The length of each bar represents the significance of that specific pathway. In addition, the brighter the color, the more significant that pathway is

Besides SAL and Enz, exosomes were isolated following treatment with two other AR-antagonists, Dar and AA. Subsequently, MS-spec analyses let to the identification of a total of 83 common upregulated proteins (Supplementary Table S9) in isolated exosomes after treatment with SAL, Enz, AA, or Dar. To elucidate the interconnections among these 83 exosomal proteins, a protein network was constructed using Cytoscape [50] (Supplementary Fig. S5). The top 10 hub-proteins are depicted in Fig. 7 A. Pathway analysis was performed by Enrichr web tool [33–35], indicating an enrichment of vesicle- mediated transport, membrane trafficking, RAB GEFs exchange GTP for GDP on RABs, and signaling by EGFR pathways (Fig. 7 B). Also these predictions suggest an upregulation of tumor promoting pathways.

### Growth promotion of LNCaP cells by treating with secreted exosomes regulated by AR-ligands

**Fig. 8:**
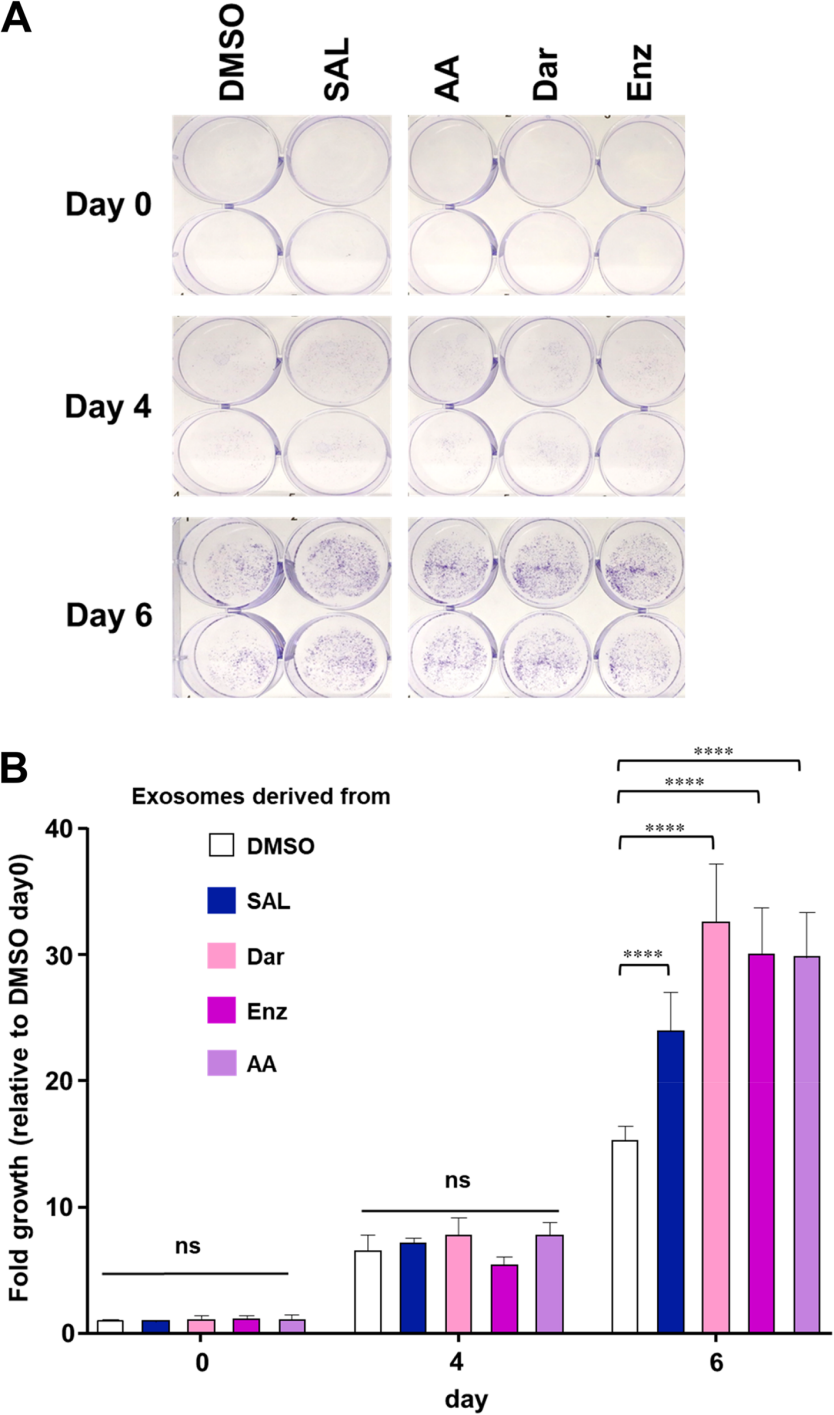
The secreted exosomes by AR-agonist and -antagonist lead to the enhanced PCa growth. **A** Representative crystal violet staining pictures of cells after 6 days of treatment. **B** Crystal violet absorbance (OD 590 nm) was normalized to the value of DMSO day 0. Bar graphs are shown as mean + SEM from total of six technical replicates of three independent experiments (*n* = 3). Statistical analysis was performed by using two-way ANOVA. (****p ≤ 0.0001)

To functionally verify the bioinformatic analysis, growth assays were conducted (Fig. 8 A). This assay evaluates the growth stimulation of AR-ligand naïve LNCaP cells exposed to the isolated exosomes derived from conditioned medium collected from cells treated with the indicated AR-ligands (Fig. 8). In addition to exosomes derived from cells treated with SAL or Enz, the treatment with the exosomes obtained from AR-antagonists, Dar and AA, were included. These were subsequently compared to cells treated with the exosomes derived from solvent control (DMSO). The data suggest a significant growth enhancement of LNCaP cells (Fig. 8 B). Enhanced growth was observed by exosomes derived from SAL and also from those derived from cells treated with the indicated AR-antagonists. The results functionally verified the bioinformatics analysis that secreted exosomes contain growth-promoting proteins regulated by AR-ligands.

## Discussion

The interplay between prostate tumor cells and their microenvironment is widely recognized as a critical determinant of disease progression. This interaction influences various aspects of PCa including survival, growth, angiogenesis, metastasis, and drug-resistance [19, 51]. Exosomes have been demonstrated to mediate tumor microenvironment communication [18].

Several discoveries support the association between cellular senescence and exosomes [52], with an enhanced exosomes secretion in response to oxidative stress and irradiation-induced cellular senescence [53–55]. However, not much is known about the therapeutically use of AR-ligands in changing exosome cargos. In light of these findings, we used AR-ligands to assess whether the AR regulates exosome protein content. Furthermore, we aimed to discern the functional consequences of the exosomes on growth. Our current data suggest a significant upregulation in the secretion of CD9, a well-established exosome marker, following SAL treatment. Conversely, Enz did not yield any discernible alterations in CD9 levels. This confirms the data published by Soekmadji et al. (2017) revealing that the secretion of extracellular vesicles upon Enz treatment is not inhibited [56]. Exosomes are known for transporting SASP factors [57]. Therefore, we analyzed TIMP2, a known SASP factor [47] in more detail. The MS-spec did not detect TIMP2 as an exosomal cargo, which is in line with the lack of co-localization with CD9. These combined results suggest that TIMP2 is not among the proteins secreted by exosomes originating from PCa cells.

In the present study, it was found that the protein expression profiles of exosomes released from AR-ligands treated cells were significantly altered. Since also AR-antagonists change the cargo of exosomes, it suggests that AR-antagonists do not solely neutralize the AR but rather activate a distinct AR signaling including the regulation of protein content of exosomes. All AR-antagonists and the use of androgens at supraphysiological level induce cellular senescence in PCa cell lines and in patient-derived prostatectomy samples shown for AA and SAL [7, 14, 20, 38]. It is possible that the AR-ligand mediated induced level of senescent cells within the cell population is one underlying mechanism of changed cargos in exosomes. Still, the different AR-ligands regulate a distinct composition of exosomal proteins. Similarly, findings by Takasugi et al. (2017) suggest that exosomes released by doxorubicin (DXR)-induced senescent RPE-1 cells (Retinal pigment epithelial cell line) resulting in a substantially altered protein composition of exosomes [52]. This suggests a possible way of how cells react under treatment by therapeutics, leading to changes in their exosomal protein content for communication within the tumor microenvironment. According to our data, an upregulation of proteins known to promote growth, including MFGE8, AKT1, AK1, CTNND1, ANXA6, CALM1, NCK1, ERBB2IP, FLOT1, FLOT2, HSPB1, PAK2, ITGA6 were detected. These proteins upregulate proliferation, migration, angiogenesis, and drug resistance [19, 58–60].

Notably, an upregulation of Ras-associated binding (Rab) family proteins in isolated exosomes were identified. This finding aligns with a prior study that illustrated the transfer of oncogenic proteins via exosomes secreted from PCa [61]. However, the effects of AR-ligands on Rab family expression were previously unclear. Here, it is suggested that AR-ligands lead to the upregulation of levels of Rab family members in exosomes. Rab family proteins are known to be involved in pro-cancerogenic pathways and impose pro-proliferative effects, further illuminating the intricate interplay between AR signaling and exosome-mediated cellular communication.

It is worth emphasizing that resistance to both first- and second-generations of AR-antagonists can develop, and one possible mechanism behind this resistance might involve the induction of cellular senescence and subsequent exosome secretion. This process can impact neighboring cells, leading to the upregulation of growth of non-senescent cells. Treating LNCaP cells with isolated exosomes derived from cells treated with AR-antagonists or SAL led to an increase in growth of PCa cells. This finding provides confirmation that the upregulation of exosomal proteins expression mediates tumor promoting and membrane activity pathways. A similar growth promoting observations following treatment with exosomes secreted from DXR-induced senescent RPE-1 cells was observed for a human ovarian cancer cell line and an oesophageal cancer cell line [52]. Our findings suggest a significant contribution of exosomes secreted from PCa cells with pro-tumorigenic activities by AR-antagonists and SAL.

## Conclusions

The evidence provided here indicates that exposure to AR-ligands induces a significant alteration in exosomal proteins released by PCa cells. These exosomes subsequently enhance the growth of LNCaP cells, highlighting a potential growth promoting activity in the tumor microenvironment. This study expands our understanding of AR controlled exosomes secretion by AR-ligands and their protein content mediating tumor growth.

### Supplementary Information

**Supplementary Material 1.**

**Supplementary Material 2.**

**Supplementary Material 3.**

## Data Availability

The datasets used and/or analyzed during the current study are available from the corresponding author on reasonable request.

## Abbreviations

AA: Atraric acid
ANG: Angiogenin
AR: Androgen receptor
BAT: Bipolar androgen therapy
Bic: Bicalutamide
Dar: Darolutamide
Enz: Enzalutamide
MS-spec: Mass spectrometry
PCa: Prostate cancer
PSA: Prostate specific antigen
SA β-Gal: Senescence-associated β-galactosidase
SAL: Supraphysiological androgen levels
SASP: Senescence-associated secretory phenotype
TME: Tumor microenvironment
