# Supplementary material for: The protein composition of exosomes released by prostate cancer cells is distinctly regulated by androgen receptor-antagonists and -agonist to stimulate growth of target cells
Source: Cell Commun Signal. 2024 Apr 8;22:219. doi: 10.1186/s12964-024-01584-z (PMC11000412; doi:10.1186/s12964-024-01584-z)
Supplement: Supplementary file 2 — Supplementary Material 2. [file 12964_2024_1584_MOESM2_ESM.pptx]

## Slide 1
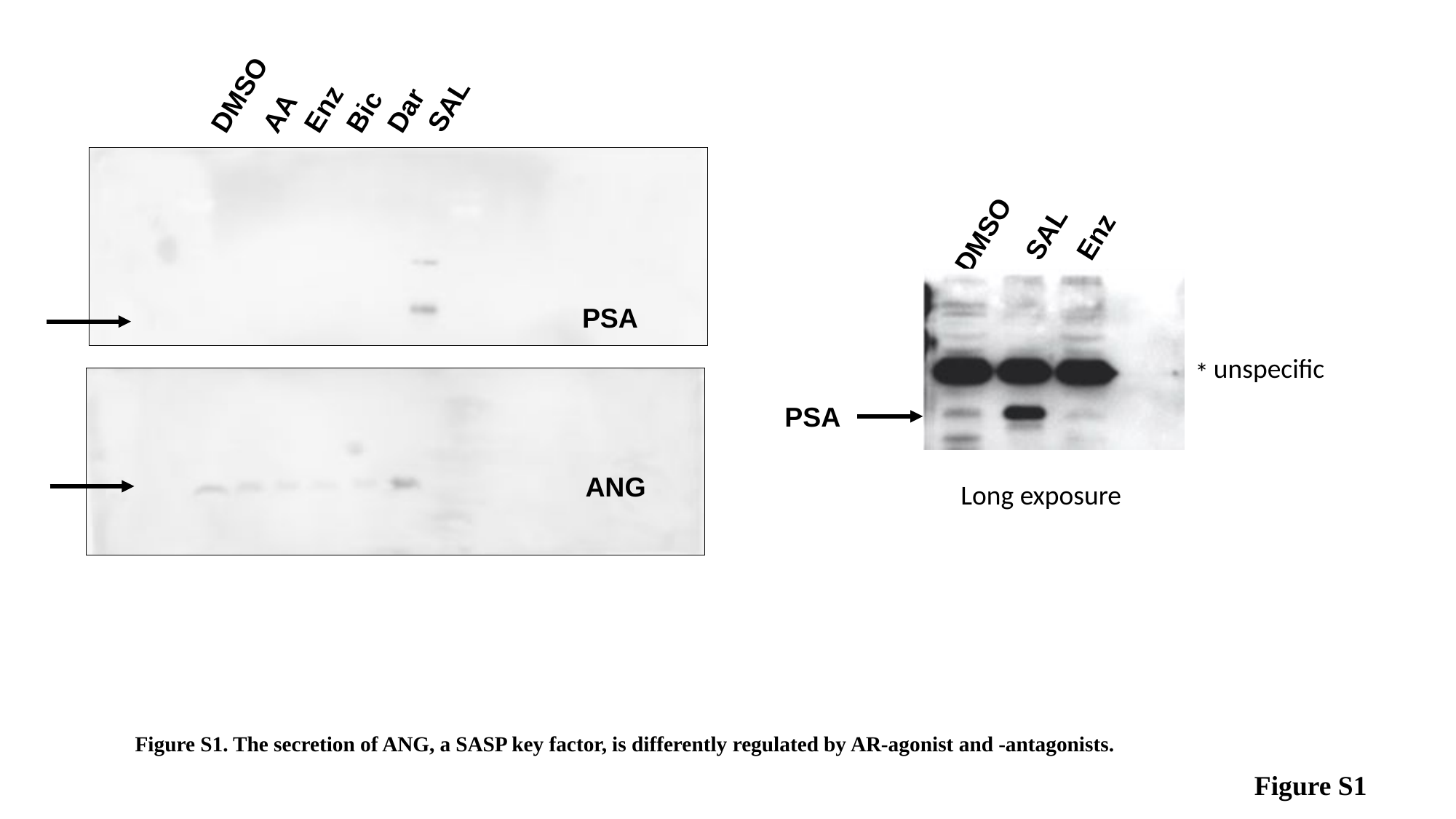

DMSO
SAL
Dar
Bic
Enz
AA
DMSO
SAL
Enz
PSA
unspecific
*
PSA
ANG
Long exposure
Figure S1. The secretion of ANG, a SASP key factor, is differently regulated by AR-agonist and -antagonists.
Figure S1
