# Supplementary material for: The protein composition of exosomes released by prostate cancer cells is distinctly regulated by androgen receptor-antagonists and -agonist to stimulate growth of target cells
Source: Cell Commun Signal. 2024 Apr 8;22:219. doi: 10.1186/s12964-024-01584-z (PMC11000412; doi:10.1186/s12964-024-01584-z)
Supplement: Supplementary file 3 — Supplementary Material 3. [file 12964_2024_1584_MOESM3_ESM.pptx]

## Slide 1
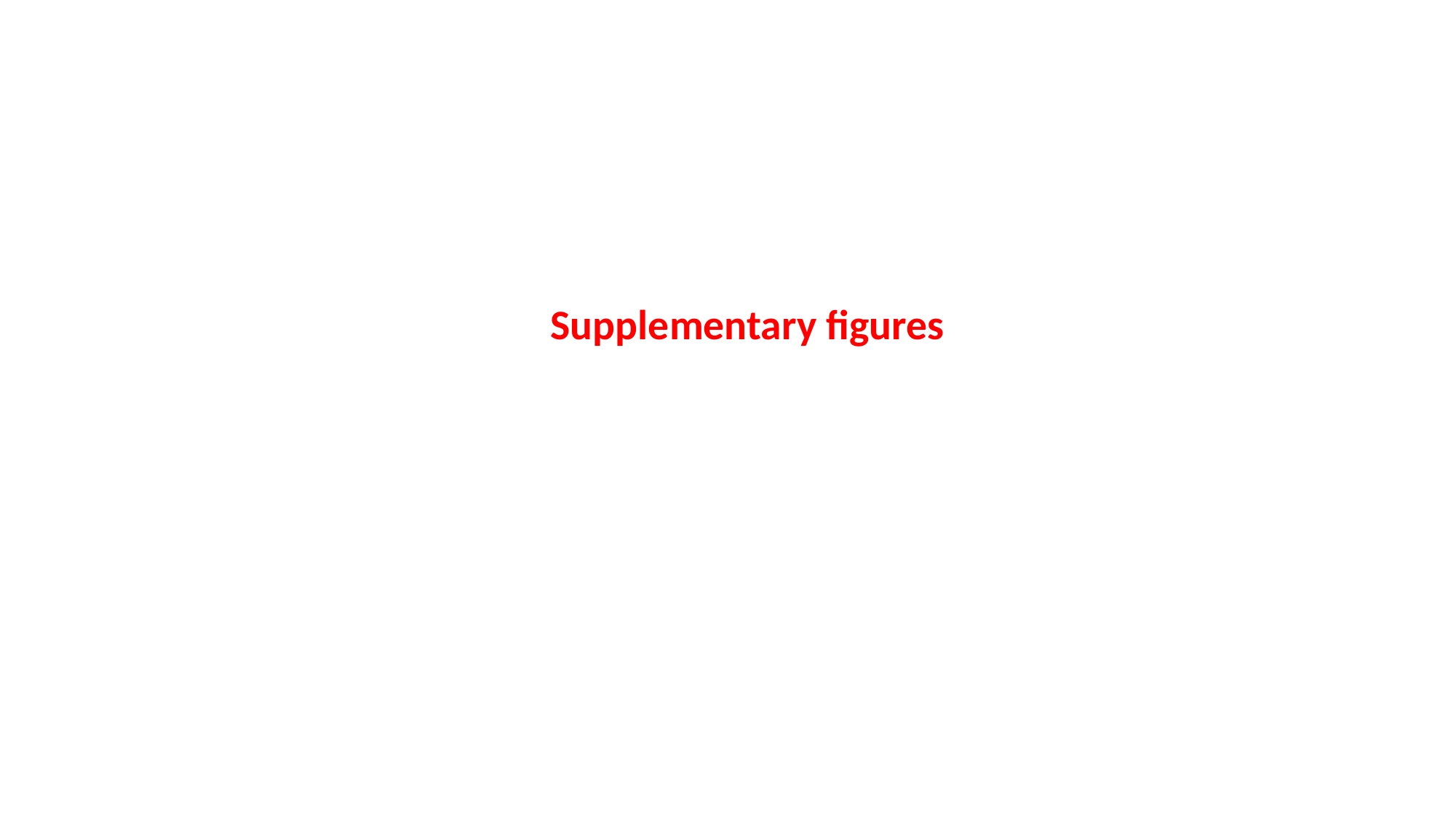

Supplementary figures

## Slide 2
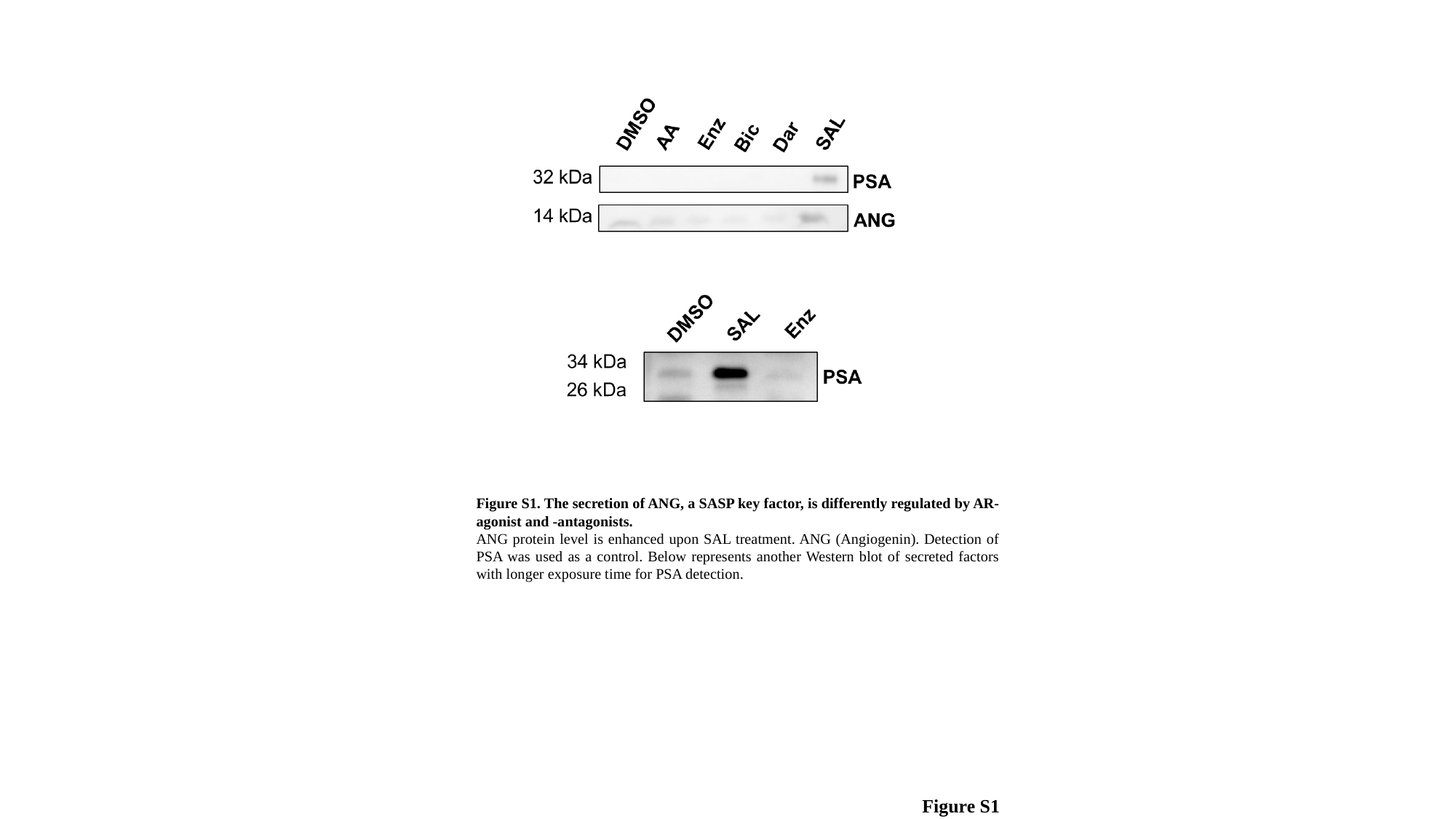

Figure S1. The secretion of ANG, a SASP key factor, is differently regulated by AR-agonist and -antagonists.
ANG protein level is enhanced upon SAL treatment. ANG (Angiogenin). Detection of PSA was used as a control. Below represents another Western blot of secreted factors with longer exposure time for PSA detection.
Figure S1

## Slide 3
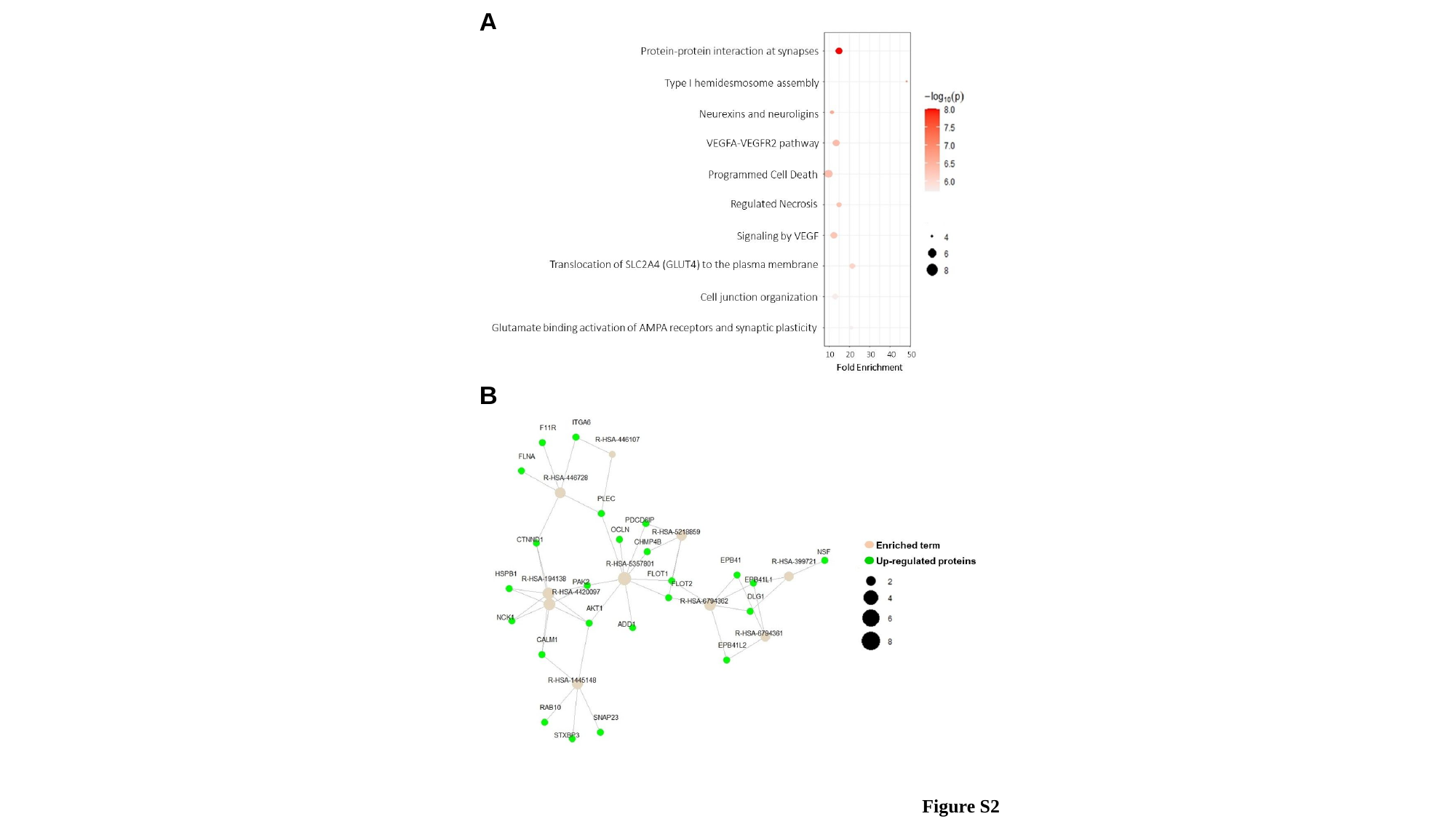

A
B
Figure S2

## Slide 4
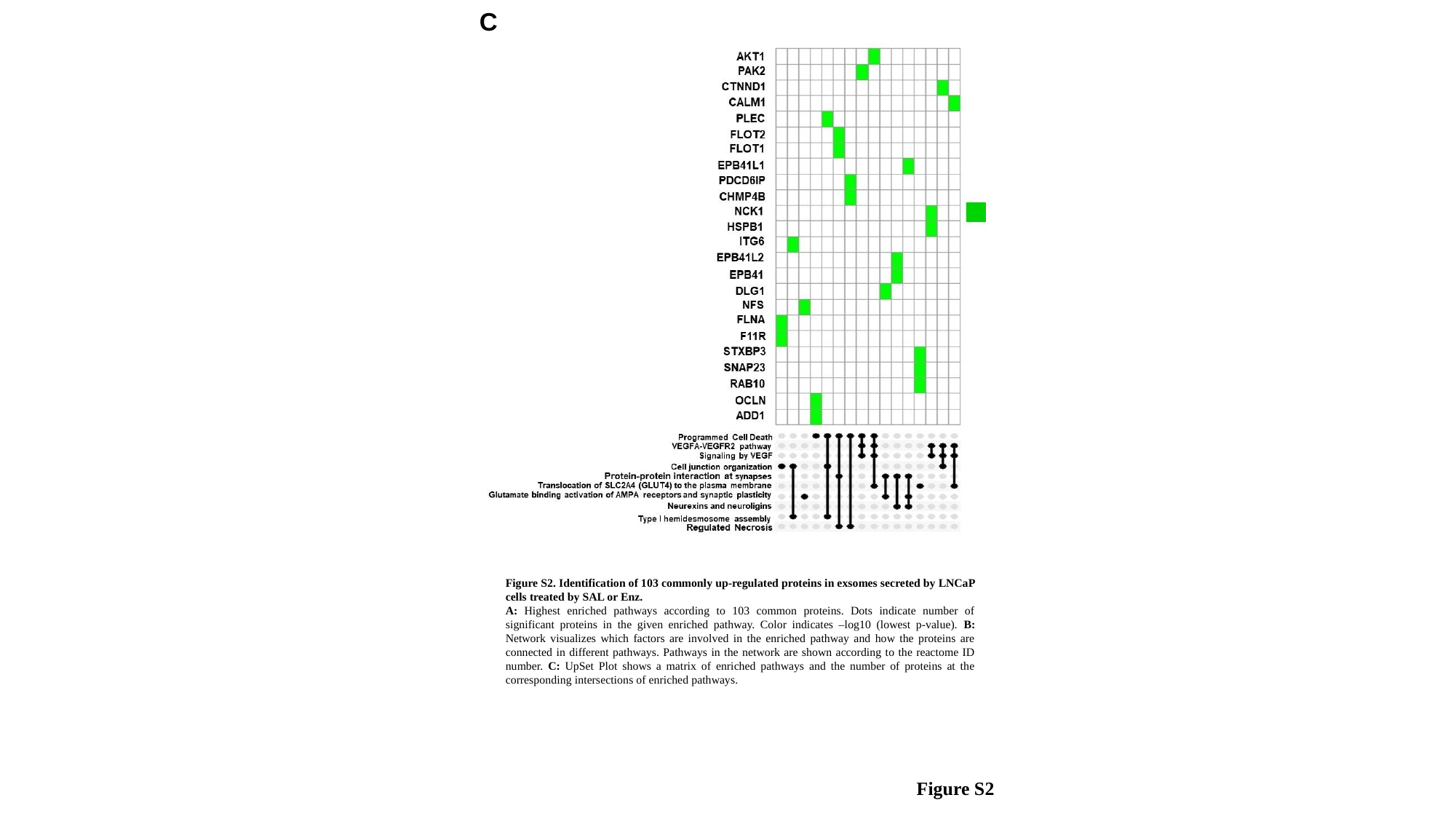

C
Figure S2. Identification of 103 commonly up-regulated proteins in exsomes secreted by LNCaP cells treated by SAL or Enz.
A: Highest enriched pathways according to 103 common proteins. Dots indicate number of significant proteins in the given enriched pathway. Color indicates –log10 (lowest p-value). B: Network visualizes which factors are involved in the enriched pathway and how the proteins are connected in different pathways. Pathways in the network are shown according to the reactome ID number. C: UpSet Plot shows a matrix of enriched pathways and the number of proteins at the corresponding intersections of enriched pathways.
Figure S2

## Slide 5
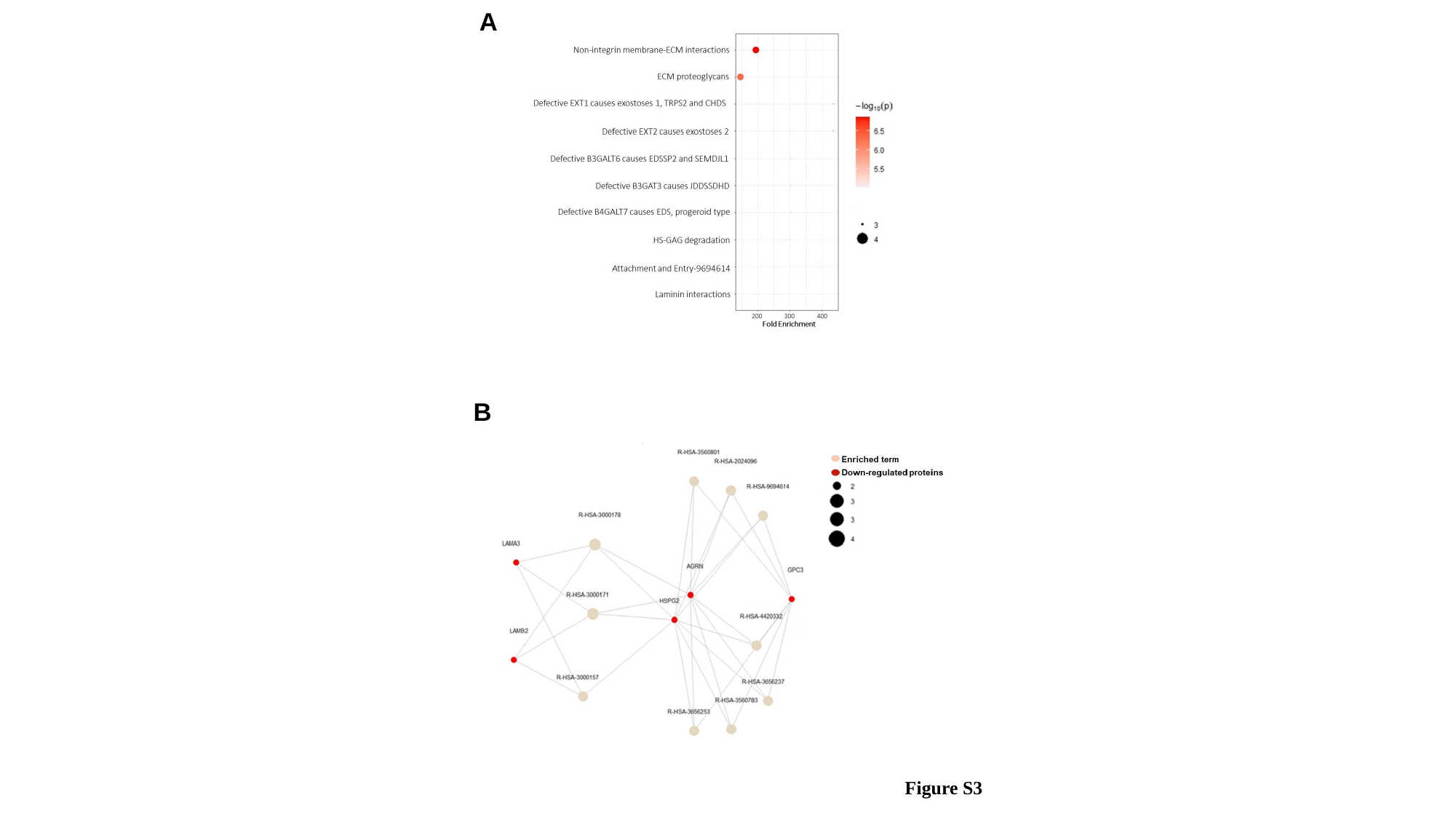

A
B
Figure S3

## Slide 6
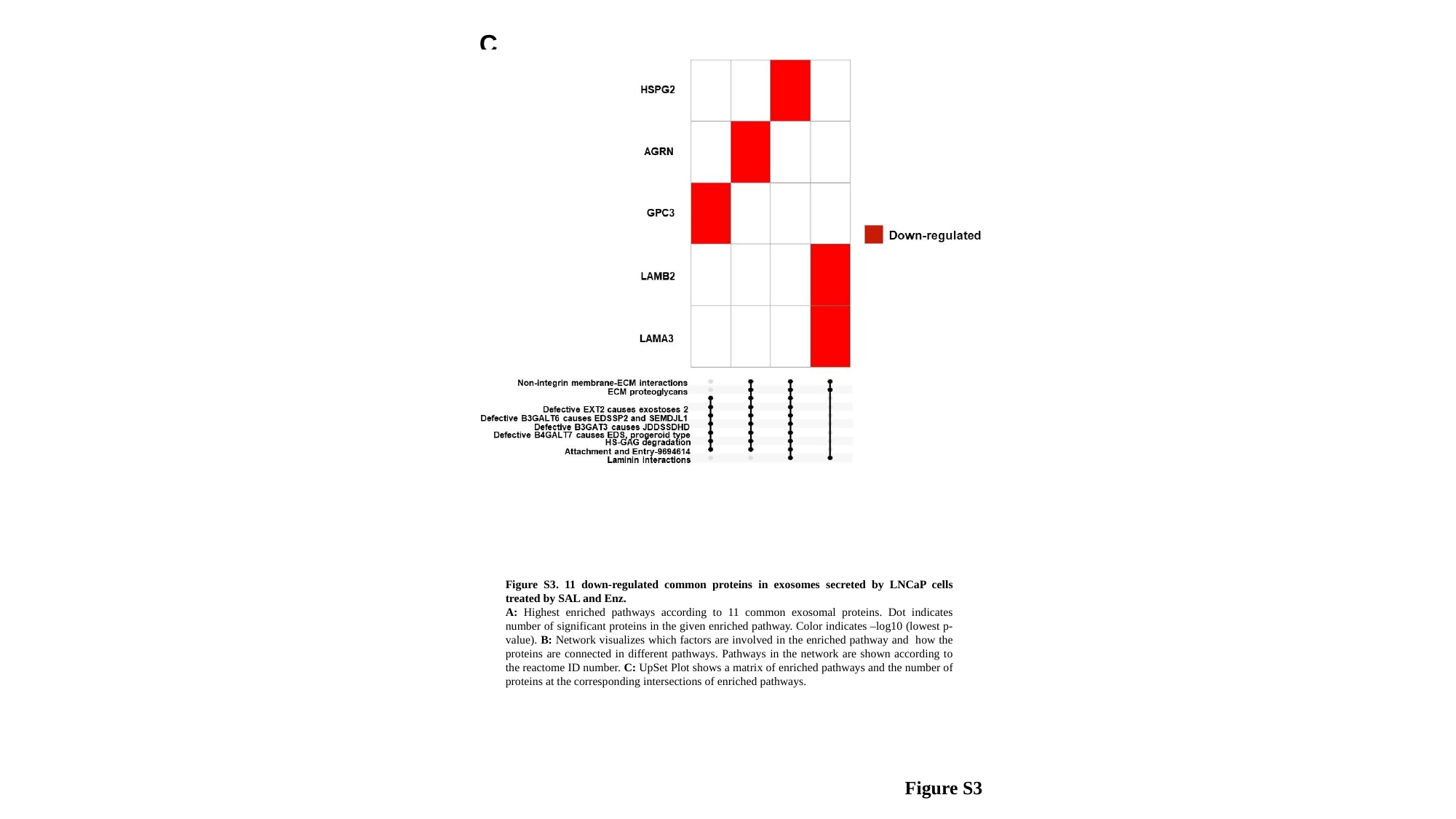

C
Figure S3. 11 down-regulated common proteins in exosomes secreted by LNCaP cells treated by SAL and Enz.
A: Highest enriched pathways according to 11 common exosomal proteins. Dot indicates number of significant proteins in the given enriched pathway. Color indicates –log10 (lowest p-value). B: Network visualizes which factors are involved in the enriched pathway and how the proteins are connected in different pathways. Pathways in the network are shown according to the reactome ID number. C: UpSet Plot shows a matrix of enriched pathways and the number of proteins at the corresponding intersections of enriched pathways.
Figure S3

## Slide 7
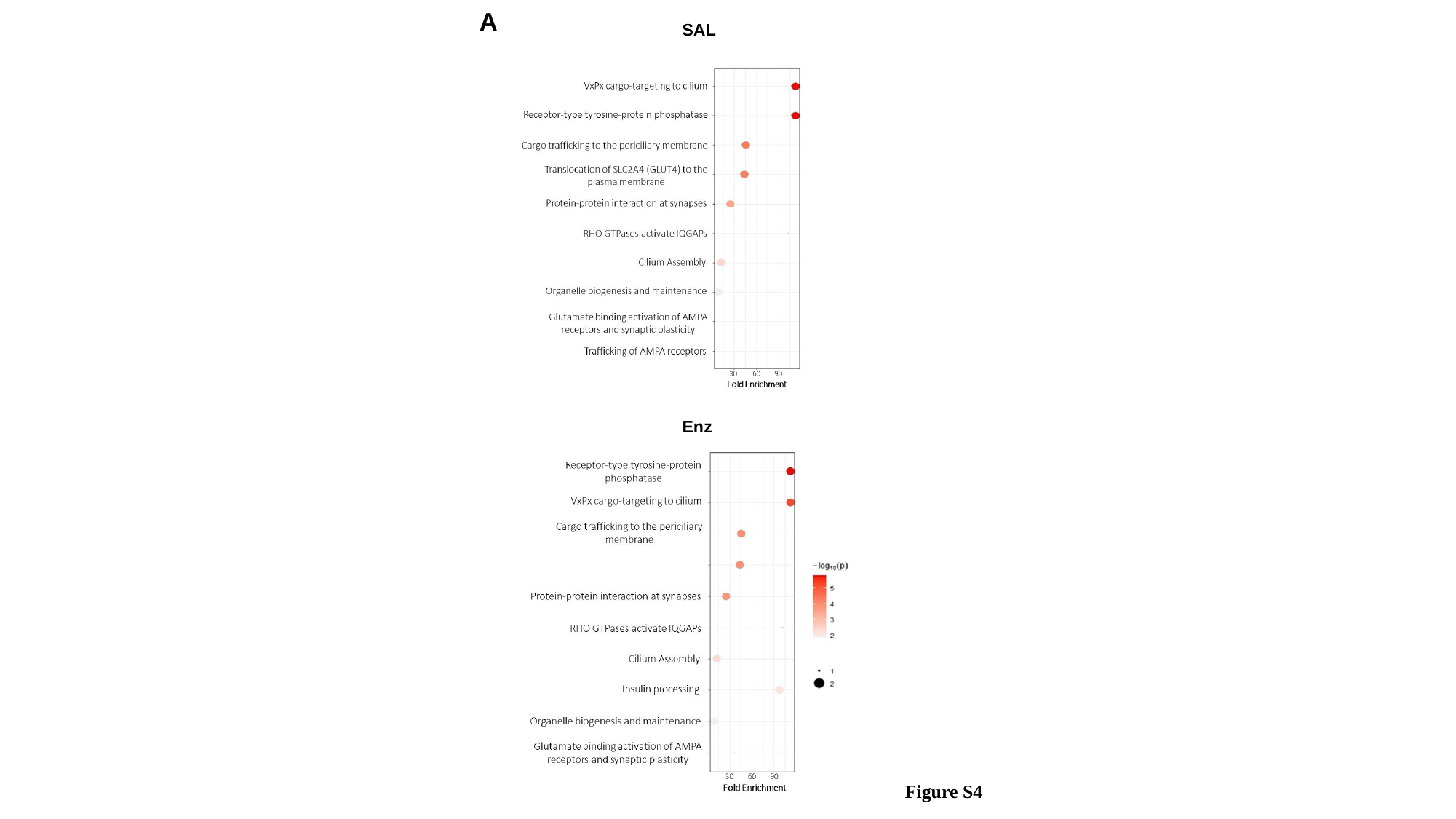

A
SAL
Enz
Figure S4

## Slide 8
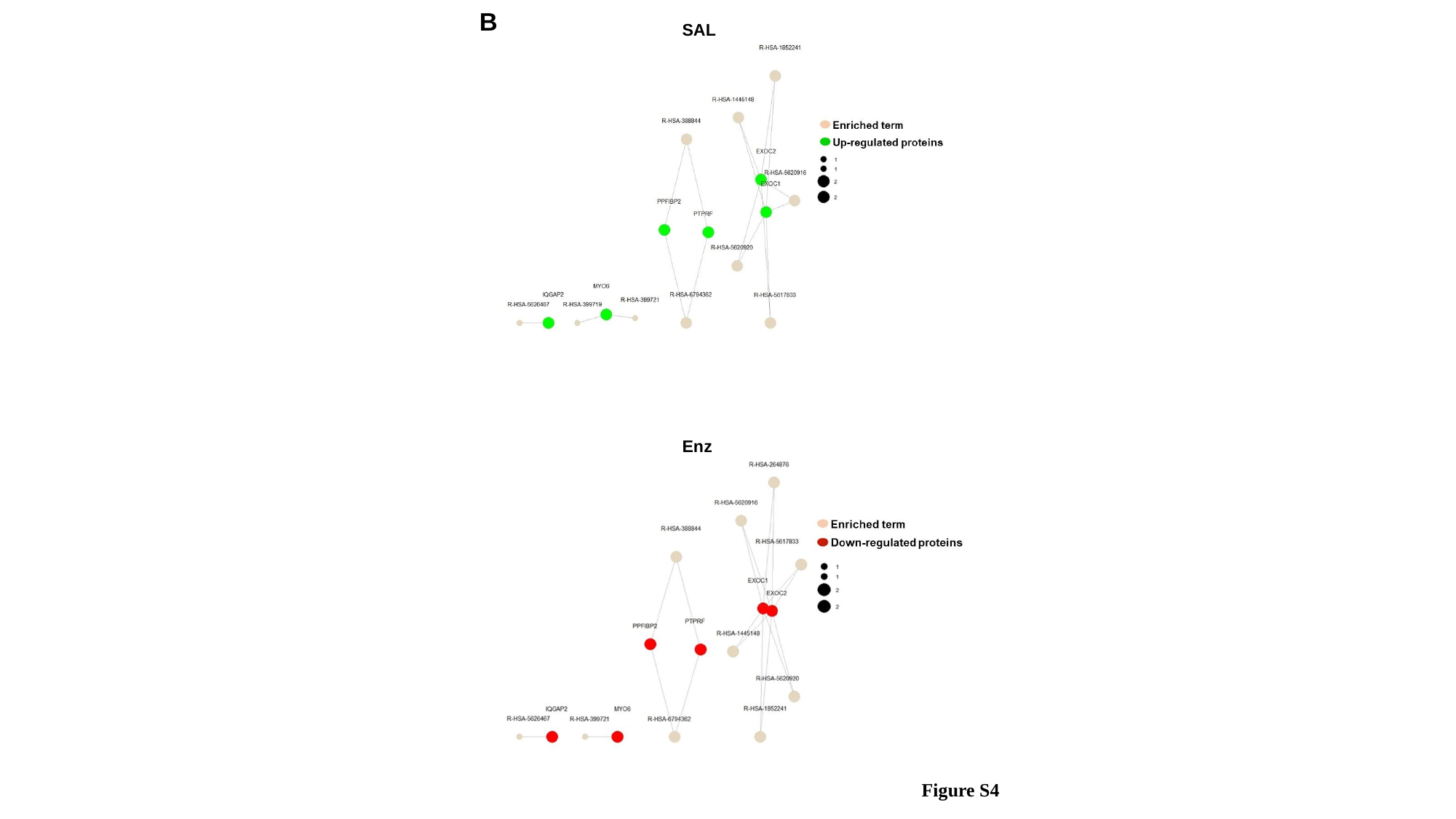

B
SAL
Enz
Figure S4

## Slide 9
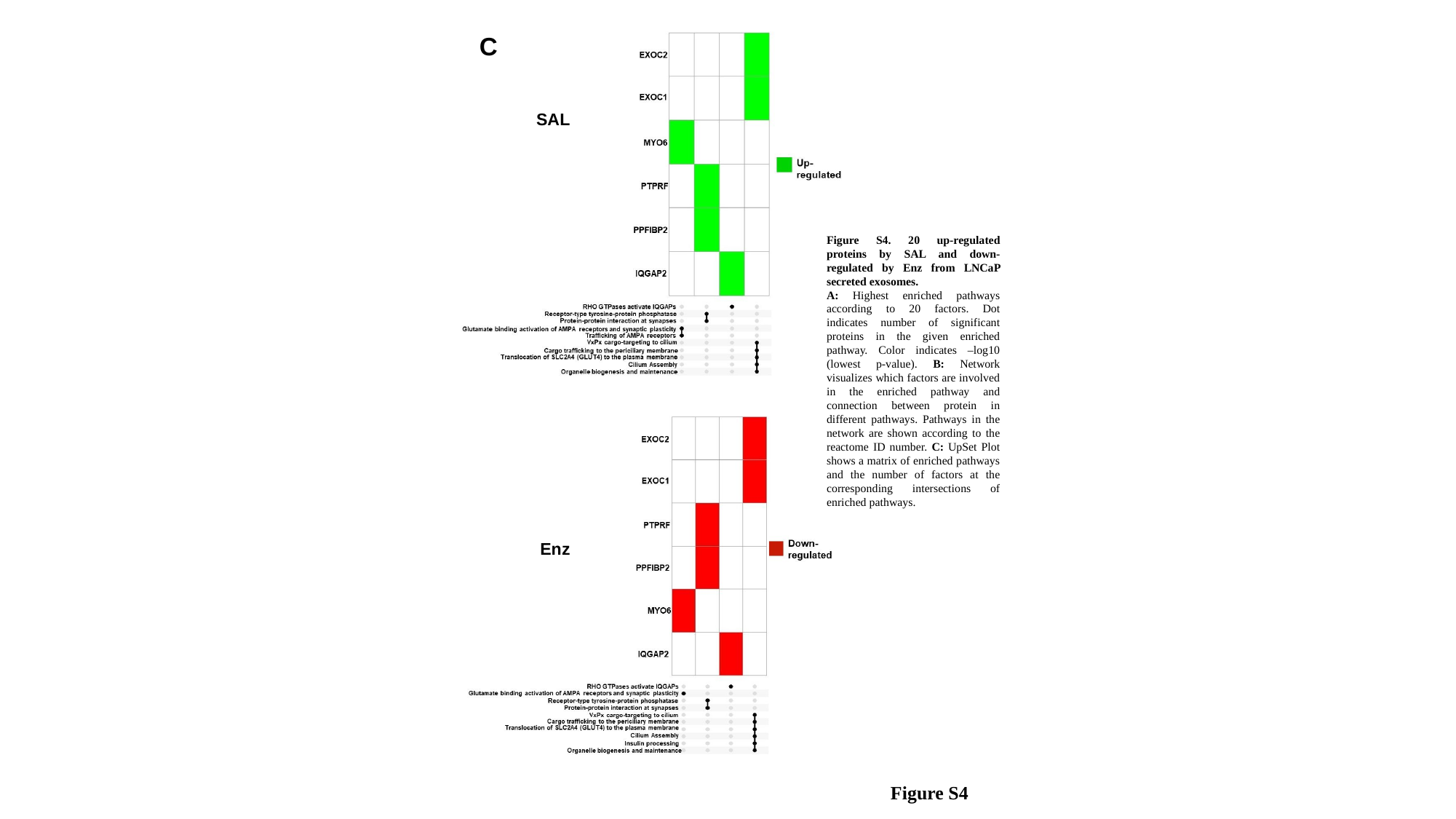

C
SAL
Figure S4. 20 up-regulated proteins by SAL and down-regulated by Enz from LNCaP secreted exosomes.
A: Highest enriched pathways according to 20 factors. Dot indicates number of significant proteins in the given enriched pathway. Color indicates –log10 (lowest p-value). B: Network visualizes which factors are involved in the enriched pathway and connection between protein in different pathways. Pathways in the network are shown according to the reactome ID number. C: UpSet Plot shows a matrix of enriched pathways and the number of factors at the corresponding intersections of enriched pathways.
Enz
Figure S4

## Slide 10
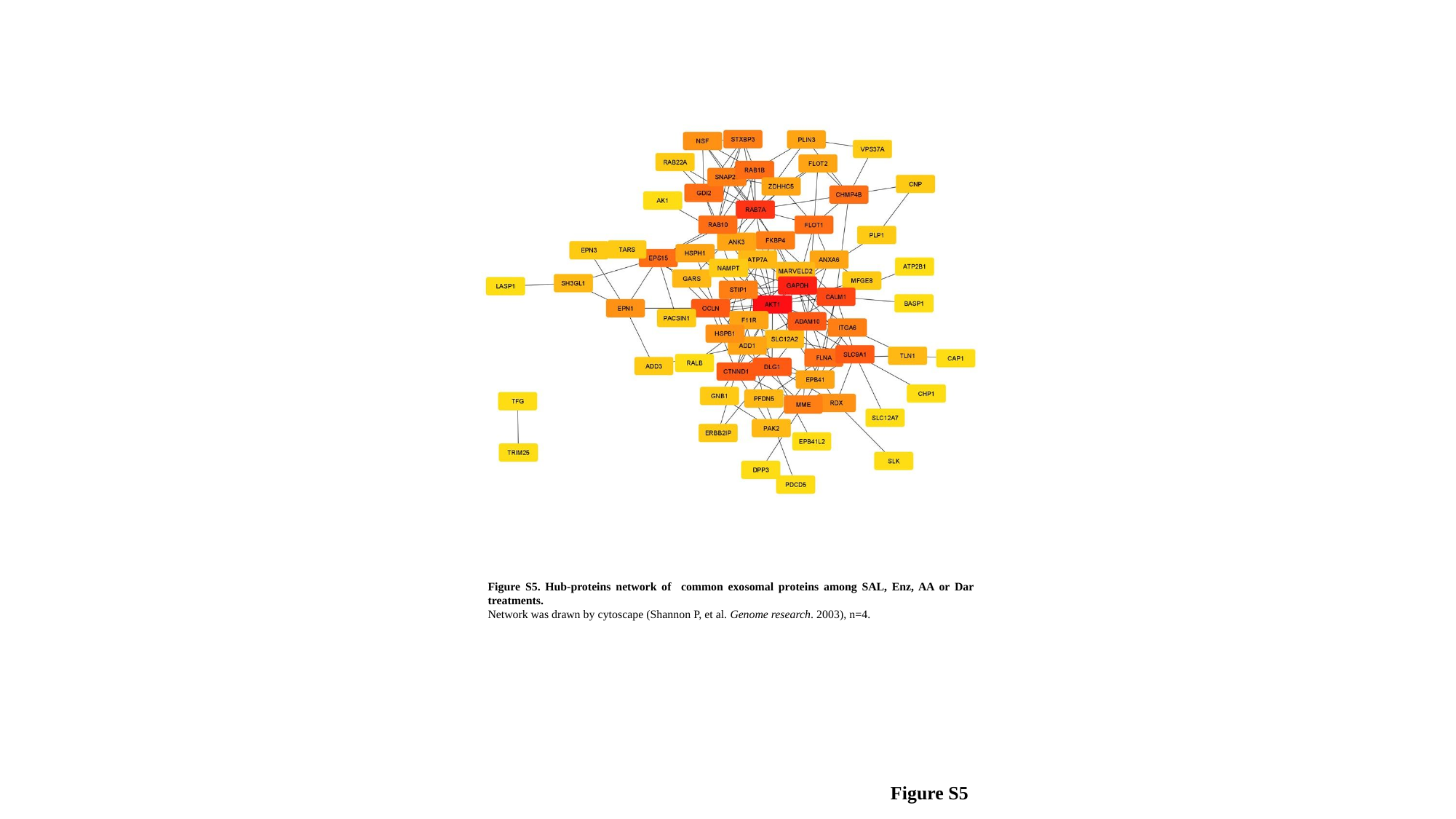

Figure S5. Hub-proteins network of common exosomal proteins among SAL, Enz, AA or Dar treatments.
Network was drawn by cytoscape (Shannon P, et al. Genome research. 2003), n=4.
Figure S5
